# Factors Associated with Perinatal Bereavement Among Mothers in Bolivia: A Qualitative Study

**DOI:** 10.3390/healthcare13060615

**Published:** 2025-03-12

**Authors:** Claudia Eva Fernández-Cox, María Fabiana Chirino-Ortiz, Tania Lara, Marion K. Schulmeyer, Manuel Fernández-Alcántara

**Affiliations:** 1 Facultad de Humanidades, Comunicación y Artes, Universidad Privada de Santa Cruz de la Sierra, Av. Trasversal 1, Santa Cruz de la Sierra 2944, Bolivia; claudiaevafernandez@upsa.edu.bo (C.E.F.-C.); mariafabianachirino@upsa.edu.bo (M.F.C.-O.); marionschulmeyer@upsa.edu.bo (M.K.S.); 2 Faculty of Health Psychology, Valencian International University, 46002 Valencia, Spain; tania.laraestepa@gmail.com; 3 Department of Health Psychology, University of Alicante, 03080 Alicante, Spain

**Keywords:** perinatal grief, mothers, support, qualitative study, stigma, bereavement

## Abstract

**Background/Objectives**: The objective of this research was to analyze the experiences and factors associated with perinatal grief in mothers in the urban context of Santa Cruz de la Sierra, Bolivia. **Methods**: The sample consisted of seven mothers who experienced a loss during pregnancy up to the second month after the baby’s birth, occurring between 2015 and 2020 in the city of Santa Cruz de la Sierra. The mean age of the mothers was 34.86 years (SD = 3.13), and they belonged to middle or upper-middle socioeconomic levels. Data were collected through semi-structured interviews and analyzed using descriptive qualitative analysis. **Results**: The identified characteristics of perinatal grief were sadness, anger, guilt, emotional numbness, social isolation, and anxiety. Factors contributing to grief processing included support from the partner and family, when they accommodated and respected the mother’s needs. Factors hindering the grieving process included social and cultural environments that often silence and minimize the loss, a history of previous losses, the desire to be pregnant, and the mother’s life expectations and projects focused on motherhood. **Conclusions**: In conclusion, this research suggests that perinatal losses in the Bolivian context may be influenced by factors such as knowledge of the cause of death, previous loss experiences, and their emotional effects. The limitations of the study include the lack of diversity in participants’ educational and socioeconomic backgrounds and the restriction of the sample to an urban area in Bolivia. Emotional interventions to support these bereaved mothers in those complex moments should be integrated in the Bolivian healthcare system.

## 1. Introduction

Perinatal grief is the experience parents go through following the loss of their child due to miscarriage, stillbirth, neonatal death, or elective termination due to fetal anomalies [1]. Perinatal grief is highly complex and distinct from other types of loss, often associated with a lack of understanding from family, social, and healthcare environments [2].

Perinatal loss or mortality encompasses the period beginning at 22 completed weeks (154 days) of gestation and ending 7 full days after birth [3]. However, in practice and previous research, this period is often extended [4]. The neonatal period begins at birth and ends 28 days after [3]. Epidemiologically, Bolivia’s perinatal mortality rate in 2018 was approximately 13.5 per 1000 live births. In Santa Cruz, the estimate is even lower, with a value of 9 per 1000 live births [5].

Perinatal grief shares many characteristics with other types of loss while also presenting unique aspects. It involves not only the loss of a loved one but also the loss of an unrealized future, the rupture of expectations, the negation of anticipation, and its impact on the couple as parents [6]. Additionally, key risk factors for developing complicated perinatal grief include lack of social support, high levels of guilt and self-blame, pre-existing psychopathology or emotional problems, as well as a previous history of perinatal losses [7].

Perinatal grief falls within the category of disenfranchised grief. Perinatal losses remain a taboo subject today [8]. These losses are socially unrecognized and invalidated, neither openly expressed by the individual nor supported by their surroundings [9]. This grief occurs in a climate of solitude due to the lack of support from family, social, and even healthcare environments. Consequently, grief is usually denied. This loss may be unacknowledged, minimized, and silenced. Such circumstances exacerbate the pain, silence, and withdrawal experienced by these mothers, often prolonging or chronicizing their grief [6].

Few theoretical models have been specifically developed to understand perinatal grief. One notable example is Marianne Hutti’s model, which explains the intensity of perinatal grief based on the reality of the loss, the congruence of the experience, and the coping mechanisms employed by mothers and fathers [10]. Additionally, the Dual Process Model, widely applied in grief research, was later revised to emphasize the central role of the family system and how each member’s coping responses influence the entire system [11].

Several qualitative studies have explored mothers’ experiences with perinatal grief. For instance, a study using interpretative phenomenological analysis on miscarriage experiences by Meaney et al. [12] highlighted the devastation parents felt upon being informed of their loss. Camacho-Ávila et al. [13] described the experiences and perceptions of parents enduring perinatal death in Spain. The grief following perinatal death began with the perception of threat and anticipation of death, linked to prior experiences, medical history, warning signs like changes in fetal movement patterns, and maternal premonitions. Confirmation of death led to emotional shock, characterized by pain and heightened feelings of loneliness.

The grieving process’s experience depends on various factors, including gestational age, knowledge of the cause of death, previous loss experiences, the bond established with the baby, and support from the partner, family, and sociocultural environment.

Concerning gestational age, Mota González et al. [14] found a positive correlation between gestational age and grief intensity; as gestation progresses, grief intensity increases. Complicated grief was more frequent in women with pregnancies lasting over 28 weeks [15]. However, this finding was not consistently replicated, as seen in the study by de Oliveira Trintinalha et al. [16], which found no significant differences in grief intensity between parents who lost a baby in the first trimester and those who lost a baby during later perinatal or neonatal periods.

Regarding knowledge of the cause of death, parents often seek reasons to assign blame or absolve themselves of guilt [17]. This search and the accompanying frustration often leads to blaming others or themselves for the loss [18]. Meaney et al. [17] noted that mothers experiencing antepartum loss focused on their behaviors during pregnancy, fearing negligence. Once parents identified a reason for their baby’s death, they felt immense relief.

In terms of prior loss experiences, Claramunt et al. [19] suggested that multiple prior losses could intensify initial grief reactions. Román Abrams and Plaza Montero [20] reported that overcoming the fear of a similar situation in future pregnancies was a significant challenge. This fear caused constant thoughts about past experiences and doubts about future pregnancies. Women also often experienced depression symptoms, avoided reminders that could trigger sadness or negative thoughts, and faced the possibility of not having more children.

With respect to the bond established with the baby, Santos Redondo et al. [21] noted that future plans, emotional bonding, and the idealization of the maternal role intensify as gestation progresses and as the desire to conceive increases. The more unexpected the loss, the more advanced the gestation, and the more desired the pregnancy, the harder it is to process and assimilate the loss. As Román Abrams and Plaza Montero [20] stated, throughout history, motherhood has been a central aspect of women’s lives. Many women link their personal fulfillment to this role and project various expectations onto their children. The perinatal stage is thus filled with dreams and aspirations tied to this idea of motherhood.

Finally, regarding perceived support, Claramunt et al. [19] asserted that if family, friends, and healthcare providers are sensitive to parents’ emotional needs, that can have a positive effect on grief intensity. This aligns with Gopichandran et al. [18], who stated that emotions after perinatal loss were intensified by an insensitive healthcare system, providers, friends, neighbors, and marital tension. Román Abrams and Plaza Montero [20] identified support during perinatal grief as a key element for women to cope with the loss, with their partners and families as their main sources of support.

Research in this field remains scarce, particularly in Latin American countries, as studies predominantly focus on nursing staff’s training needs and their roles. Few investigations examine mothers’ grieving processes, with most focusing on coping strategies and psychological aspects like self-esteem, resilience, and social support, mainly through quantitative studies or systematic reviews. A review of major academic databases confirms the absence of scientific research in the domain of grief in Bolivia. Recently, only two multicenter studies have addressed pandemic grief symptoms, as well as suicidal ideation, but no studies were found regarding the emotional experience of bereavement [22,23]. This highlights the importance of further exploring the experiences of mothers who endure perinatal loss and the factors associated with perinatal grief.

Thus, the main objective of the present research was to delve deeply into the experience and factors associated with perinatal grief in mothers in the urban context of Santa Cruz de la Sierra, Bolivia. The research questions were the following: What is the experience of perinatal grief for mothers of babies born in Bolivia? What are the factors that influence the experience of perinatal grief in this population?

## 2. Materials and Methods

### 2.1. Design

This study employed a descriptive qualitative design. Hernández and Mendoza [24] defined qualitative methods as those that try to understand the phenomena, exploring them from the perspective of the participants in their natural environment and in relation to the context. A qualitative method was used, due to the nature of the study, as it focused on deeply understanding the experiences of mothers who suffered a perinatal loss. According to the evolution of the research, it was focused on the various factors associated with perinatal grief. Therefore, it is a descriptive study where the researcher selects a series of questions and information is collected about each of them in order to characterize what is being investigated [24].

### 2.2. Participants

Purposive sampling was used to select women who had experienced perinatal loss in Santa Cruz de la Sierra, Bolivia. Through four key informants, contact was made with the mothers interviewed. The sample consisted of seven women who had experienced perinatal loss (see Table 1). The inclusion criteria for this study were as follows: (a) losses occurring from conception until the second month after the baby’s birth, and (b) losses that occurred between 2015 and 2020 in the city of Santa Cruz de la Sierra.

Sociodemographic information is included in Table 1. The mothers’ ages ranged between 30 and 39 years (Mean age = 34.86 years; SD = 3.13). All participants were employed and reported belonging to a middle or upper-middle socioeconomic level. Additionally, all women were in a relationship. The time of loss ranged from 11 weeks of gestation to 2 months postpartum. None of the mothers had a prior psychiatric history.

### 2.3. Instruments

A semi-structured interview was designed for data collection based on the review of various scientific articles. The questions were formulated based on theoretical frameworks suggesting that various factors influence perinatal grief experiences (see Table 2).

### 2.4. Procedure

Participants were contacted via telephone to schedule a date for the interview. They were informed about the objectives of the research and assured that the information provided would be protected and used exclusively for research purposes. Subsequently, interviews were conducted via Zoom or in person, depending on each mother’s availability. The interviews were audio-recorded and then transcribed. The project was approved by the Ethics Committee of the responsible university (Reference: UA-2020-03-11).

Before starting the interviews, the objectives of the research were explained to the participants, so they could ask any question about their participation. To protect participants’ confidentiality, codes were used instead of their names. Furthermore, before interview recording began, their verbal and written consent was requested and signed. The research team had no previous relationship with participants. The interviews lasted an average of 62 min. It should be noted that there was no link between the researchers and the participating mothers.

### 2.5. Data Analysis

The data systematization and analysis involved transcribing the interviews and organizing the information into units, categories, themes, and patterns [25]. Interpretation was conducted through interpretative triangulation, linking the data generated by the applied techniques with pre-existing theories and researcher reflexivity. This approach identified general themes, improving the understanding of experiences and factors associated with perinatal grief.

Despite the sample size, the analysis reached data saturation in the main themes and codes. Given that participants no longer provided new information and the same themes were repeated, authors decided to conclude data collection.

To guarantee the rigor of the analysis, triangulation among researchers was used during the process of analysis. The first author developed the initial codes for the analysis. Later, the codes and main themes identified were reviewed and approved by consensus among the four members of the research team.

## 3. Results

The findings were presented in line with the research objectives, addressing contextual aspects of perinatal loss, emotions associated with the loss, bonding with the baby, and support and silence in the face of loss.

### 3.1. Theme 1. Contextual Aspects of Perinatal Loss

The contextual aspects of perinatal loss identified included gestational age, knowledge of the cause of death, history of previous losses, and their emotional effects. These factors had a significant impact on the women, revealing that specific differences and particularities influenced the perinatal grief process (see Table 3).

The gestational age was linked to the events that occurred at different stages of pregnancy when it ended. This could involve a challenging labor, a surgical procedure, or, in some cases, the natural completion of the pregnancy. Regarding gestational age, early losses were just as painful as late ones. From the participant discourses, it seems that trauma could arise at any stage of pregnancy, being more related to the procedure of removing the baby rather than the gestational age itself.

For early losses, medical staff often did not provide mothers with a clear explanation of the baby’s cause of death, instead simply describing the baby’s condition at the time. As a result, many mothers remained unaware of the exact cause of their loss. In contrast, with later losses, there was often a clear cause of death, and medical professionals were more willing to conduct an autopsy. However, when the loss was unexpected, it had a greater emotional impact on mothers, often leading them to blame the hospital for the outcome.

Two of the seven interviewed mothers experienced multiple losses. Participant P5 went through three losses, while participant P6 experienced two. For both mothers, the first loss did not have a significant emotional impact. P5, for example, had planned to space her children further apart and already had an 11-month-old baby. P6, on the other hand, barely mentioned her first loss, which was a spontaneous miscarriage at five weeks following the removal of an intrauterine device, focusing instead on her second loss. Both described their losses as traumatic, and stated that recurrent miscarriages/pregnancy loss fostered ambivalence to getting pregnant again shortly after their loss.

The sense of emptiness caused by an interrupted maternity project was often heightened or brought back into focus by the presence of objects that reminded the mothers of the pregnancy, loss, and absence. For this reason, four mothers chose to store away their baby’s belongings to avoid seeing them. At the same time, the need to keep certain objects connected to the baby was observed, reflecting the difficulty of separation, a common experience in the early stages of grief. Another significant effect of perinatal loss was the lingering fear that manifested in subsequent pregnancies. Four mothers who became pregnant after their loss experienced anxiety and fear of a repeat occurrence, leading some to delay new pregnancies.

### 3.2. Theme 2. Emotions Associated with the Loss

With respect to the emotions experienced by the mothers, sadness was reported by all participants. Three mentioned feelings of anger, one experienced anxiety, two isolated themselves from their environment, three felt guilt, two expressed envy and rejection toward pregnant women in their surroundings, and one reported emotional numbness (see Table 4).

### 3.3. Theme 3. Bond with the Baby

This emotional bond was related to the subjective moment the mother experiences during pregnancy, the expectations she has, and the life project tied to the arrival of the child (see Table 5). All these aspects will influence how a woman experiences perinatal loss and develops coping strategies.

In terms of the timing of the pregnancy, it can be stated that, in the interviewed cases, the pregnancies, although not all planned, were desired, as the possibility of having a child was discussed at some point within the couple.

About the mother’s expectations with the pregnancy and the bond with the baby, it can be mentioned that, being desired pregnancies, expectations manifested from the beginning of the gestation. These expectations included excitement and joy, followed by buying things for the future baby, choosing names, imagining how the baby would be, inter alia. In cases where problems were detected during the pregnancy, there might have been a delay in announcing the pregnancy, and the mother may have tried to avoid forming expectations. However, if the pregnancy was later declared viable, the mother began to create hopes and dreams.

Regarding the mother’s life project, the perinatal loss led three mothers to try becoming pregnant again, and upon succeeding, their life project once again centered on motherhood. For another mother, motherhood was set aside after the loss; the love she felt for her daughter could not be given to another baby. Although her husband considered adoption, it is not an option for her yet. She still feels the void left by her daughter, which prevents her from projecting toward the future. Two of the interviewed mothers focused on new projects. Interviewee P5 shared a joint project with her partner that they are very excited about. Interviewee P6 is focused on professional growth and raising her son. For one mother, aged 34, motherhood has become a significant part of her life project, as she has already started paying attention to her diet to try again next year.

### 3.4. Theme 4. Support and Silence in Face of Loss

This section analyzed the role of the family, partner, social environment, and cultural context in the grieving process (see Table 6).

The role of family, partner, and social environment in the grieving process following perinatal loss varies among the interviewed mothers, demonstrating both strengths and limitations in the support provided.

The family played a significant role by offering distraction, comfort, and affection, as well as serving as emotional support. However, insensitive comments or a lack of understanding from some family members negatively impacted the grieving process for certain mothers. Sensitivity and empathy within the family environment appear to be key factors in ensuring this support is beneficial.

Partner support was identified as crucial by all interviewees. This relationship not only provided emotional support but also the opportunity to share the loss experience, creating a bond of closeness that was essential for coping with grief. However, differences in how each partner experienced the loss posed challenges, requiring mutual acceptance and respect. For some mothers, taking on the role of supporting their partner strengthened their relationship and enabled them to face the situation together.

The social environment, in general, maintained a stance of distance or silence surrounding the loss. This was partly due to the mothers’ decision not to share their experience and, in other cases, to the social environment’s discomfort in addressing the topic. Except for two cases, the social environment was not perceived as a fundamental source of support, highlighting the need to raise awareness in society about how to handle perinatal loss to prevent the emotional isolation of affected mothers.

In summary, family and partner support emerged as essential pillars, while the social environment, although present in some cases, requires greater awareness if it is to provide effective assistance.

## 4. Discussion

The aim of this study was to analyze the experiences and factors associated with perinatal grief in mothers from Bolivia. The main findings reveal that, regardless of the timing of the loss, all mothers experienced a significant grieving process following the loss of their baby. Mothers who experienced a loss during the first trimester were unaware of the cause of death, while those who lost their babies shortly before or after labor were informed of the cause. Feelings of grief appear to intensify when the mother faces another perinatal loss. Furthermore, the loss is accompanied by painful experiences such as putting away the baby’s belongings, which is the case for four mothers. In keeping with previous research on grief following perinatal loss [6,26], mothers also reported experiencing a range of emotions, including sadness, anger, guilt, emotional numbness, social isolation, and anxiety. It is important to highlight that all participants expressed sadness; however, other emotions were only present in some of them. The experience of losing a baby is influenced by the expectations and plans associated with the pregnancy, especially if it was desired. Although family and partner support are key, the minimization of grief by the social environment may hinder the grieving process. Despite this, many women find strength in their partners or in the possibility of attempting motherhood again.

In this study, regardless of gestational age, all mothers considered their experience traumatic, independent of the procedures involved or the stage of pregnancy. In early losses, mothers did not seek the cause of death, implicitly accepting that there was no clear cause, and their discourse did not focus on emotions such as guilt. This differs from findings by Meaney et al. [17], who observed that parents often seek the cause of death to blame someone or to absolve themselves of guilt. However, in the case of a sudden death near delivery, mothers are more likely to blame the healthcare system, particularly when they perceive inadequate care from the hospital. This aligns with Gopichandran et al. [18], who argued that frustration stemming from the search for a cause often leads to blaming others for the baby’s death. In this regard, guilt is a significant risk factor for developing complications in perinatal grief [7,27].

For mothers who experienced multiple losses, the initial reactions tended to be harsher, as the pain became more acute with each recurrence of the traumatic event. One mother mentioned being unable to endure another loss, consistent with Claramunt et al. [19]. The repetition of a traumatic event caused ambivalence in one mother, torn between wanting and not wanting to try for another baby [20].

All mothers reported feelings of pain and sadness after their loss. This aligns with the analysis of miscarriage experiences by Meaney et al. [12], which highlighted the devastation mothers felt when informed of their miscarriage. Camacho-Ávila et al. [13] reported that confirmation of the baby’s death led to emotional shock, characterized by pain and heightened feelings of loneliness. However, this was not entirely reflected in the present study, as mothers did not report emotional shock or intense feelings of loneliness.

Focusing on expectations, Santos Redondo et al. [21] found that the development of future plans, emotional bonding, and the idealization of the maternal role intensify as pregnancy progresses and the desire to conceive increases. This study concurs with the previous findings, in that future projection and emotional bonding with the baby are stronger when the desire to conceive is greater. However, these expectations can also be intense early in pregnancy and are not necessarily tied to gestational progression.

The literature suggests that the less expected the loss, the more advanced the pregnancy, and the more desired the pregnancy, the harder it is to comprehend and accept what has happened [21]. However, the current study found that, in most cases, women lost their babies unexpectedly and did not report difficulty in accepting their babies’ deaths. Additionally, all pregnancies were desired, but this did not cause significant challenges in accepting the loss. Nonetheless, the present study highlights the importance of paying attention to early gestational losses, which are often underestimated both by the social environment and research [27,28]. According to this, a qualitative study in South America showed that early perinatal losses are also associated with profound feelings of grief (such as sadness, fear and frustration) [29], as well as feelings of fear for the next pregnancy and difficulties with their husband. Moreover, the disenfranchisement of perinatal grief has motivated a new approach to research in which perinatal losses are considered as a stigmatized phenomenon [30,31], characterized by silence, minimization, loss of support from friends, family or healthcare professionals or feelings of shame and blame [26,32]. Interestingly, a recent study found that stigma experiences of bereaved parents (measured by the Stillbirth Stigma Scale) may be more intense in early perinatal losses [33]. In this regard, specific quantitative measures of grief intensity and stigma would be valuable for future research.

In this study, the partner played a significant role in the mother’s grieving process, serving as her primary source of support. This result is consistent with the literature focusing on father’s grief following a perinatal loss. Existing research carried out in different countries has shown that fathers often assume the role of supporting their partners during bereavement [34,35,36]. Additionally, mothers assumed the role of supporting and accompanying their partner, given the differences in how they experienced grief. Family support also played a key role when it adapted to the mother’s needs. These findings are consistent with Román Abrams and Plaza Montero [20], who highlighted the importance of support received during perinatal grief, with partners and family as the main sources of support. However, the family can negatively influence grief when hurtful comments fail to provide comfort. The social environment was distant, as mothers often withdrew, and the social environment avoided discussing the topic, making it an ineffective source of support, with the exception of two mothers who did receive support from their friends. One mother emphasized the insensitivity of doctors, citing a lack of empathy and offensive comments. As Gopichandran et al. [18] observed, emotions following perinatal loss are exacerbated by an insensitive healthcare system, healthcare providers, friends, neighbors, and tense marital relationships.

The Grief Intensity Model proposed by Marianne Hutti identifies three main factors influencing the experience of perinatal loss: (a) the reality of the loss, (b) the congruence between the personal experience and the perception of the loss, and (c) the coping strategies employed [37]. This model explains the bond between the baby and the mothers, as all participants perceived the loss as real, reflecting a strong emotional connection to their baby. This was evident in their actions, such as informing family members about the pregnancy, choosing baby names, and feeling joy and excitement upon learning of the pregnancy. Additionally, there was coherence between their personal experiences and their perception of the loss, as they had no difficulty accepting it and received support from their families during their grieving process. According to this model, while the loss was a profound reality for these mothers, the other two variables may have acted as protective factors against more intense symptoms and experiences of grief.

Although the present study did not explore in depth the different coping strategies used by the participants, previous research in other contexts highlights their usefulness in processing perinatal grief. For instance, religiosity and spirituality can aid in overcoming grief [18,38]. Gopichandran et al. [18] also reported other effective coping strategies, such as isolation, immersion in work, maternal love for other children in the family, social support, and hope for a future pregnancy.

This study has several strengths. It is among the first to address the experience of perinatal grief among Bolivian mothers. While grief is a universal and complex experience, it is closely tied to the cultural context in which individuals live [32,39]. In Bolivia, perinatal loss remains a taboo, often minimized, and the mother’s grief is frequently overlooked. Women may be told that they can have another child, a statement that can invalidate their grieving process. Additionally, some families and friends choose not to discuss perinatal loss, believing that doing so would cause the mother further distress. This lack of recognition of perinatal grief often results in mothers navigating this experience in isolation [8]. As we have previously suggested, stillbirth appears to be a disenfranchised loss, as shown by research conducted in various countries worldwide [26].

Furthermore, healthcare services are not always equipped to provide adequate emotional support for women who have experienced such a loss. However, some mothers find solace and acceptance through their religious beliefs. Nevertheless, they may not always be able to perform rituals to bid farewell to their baby, particularly in cases of early pregnancy loss, where the burial of the baby’s body is not possible. Previous research has indicated that healthcare providers may face important emotional obstacles when supporting bereaved families. Feelings of sadness, anguish, helplessness, and avoidance behavior have been reported after a perinatal loss among professionals working in hospitals [40]. Understanding the emotional reactions of mothers after a perinatal loss may help healthcare providers and give them some context to perform their interventions and follow up on the grieving process.

This study provides novel insights into the experiences of Bolivian mothers who have suffered perinatal loss, opening the door for future research aimed at deepening understanding, establishing specialized clinical care protocols, and promoting actions to raise awareness and improve social support. It emphasizes that support from various social spheres, such as the community and healthcare system, is an essential protective factor in coping with perinatal loss [6,26].

This study has several limitations. First, the sample lacked diversity in terms of participants’ educational levels, socioeconomic conditions, and occupational roles (e.g., homemakers versus employed mothers). Additionally, no interviews were conducted with mothers who experienced multiple pregnancies with one baby surviving or cases of babies born with malformations. Second, the sample was limited to an urban area in Bolivia, excluding participants from other geographic regions who might contribute complementary perspectives.

Future research is needed including the discourse of participants with diverse educational, socioeconomic, and occupational backgrounds, as well as mothers who experienced multiple pregnancies. Additionally, expanding the study to rural and other regions of Bolivia could provide a more comprehensive understanding of perinatal grief in this country. Finally, the comparison with other Latin American countries regarding the experience of perinatal grief and stigma may provide further insight into cultural similarities and differences, shedding light on shared challenges and potential areas for intervention.

## 5. Conclusions

In conclusion, this study highlights that perinatal loss in Bolivia is shaped by factors such as the cause of death, prior loss experiences, and contextual events surrounding the loss. Regardless of gestational age, grief is marked by emotions like sadness, guilt, and isolation, influenced by social and cultural minimization. While family and partner support play a key role in coping, some mothers find resilience in their relationships or the hope of future motherhood.

Considering these findings, it is essential for Bolivia’s healthcare system to implement support protocols for both early and late perinatal losses. Providing guidance that helps process the complex emotions associated with loss, along with a nonjudgmental space, appears highly beneficial in cases of perinatal grief [41]. Additionally, specific interventions that facilitate memory-making or bonding with the baby may be useful, as long as they help give meaning to the loss and counteract the taboo and silence surrounding infant death. Finally, it is important to promote some of the coping strategies highlighted in this study, such as support from partners and the creation of new meanings after the loss. Involving men in these processes and addressing their needs and experiences related to perinatal grief can be highly beneficial for both partners.

## Figures and Tables

**Table 1:** Sociodemographic data of participants.

Code	Age	Occupation	Time of Loss	Moment of Loss
P1	37	Commercial Engineer	4 years	16 weeks of gestation
P2	35	Doula and Lactation Consultant	1 year	11 weeks of gestation
P3	32	Organizational Psychologist	2 years	11 weeks of gestation
P4	30	Psychologist	1 year	2 months postpartum
P5	39	Graphic Designer, Marketing and Advertising Graduate	1 year	12 weeks of gestation (last of three losses)
P6	37	Veterinarian	8 months	36 weeks of gestation (last of two losses)
P7	34	Lawyer	2 months	12 weeks of gestation

**Table 2:** Interview script.

Interview Questions	Areas Assessed
1. How would you describe the moment when pregnancy occurs?	(a) Moment in your life: choice or contingency (b) Moment for the couple: possibility, decision (c) Desires/expectations with this pregnancy (d) Effects on your life of pregnancy and loss
2. How could you describe the experience of losing your baby?	(a) Type of loss, cause (b) Event: situation or circumstance (c) Time at which the loss occurs: intrauterine or born (d) Other experiences of loss
3. How could you describe the experience after the loss?	(a) Farewell rites (b) Who accompanied her and how they did it (c) What professional support were you offered and/or sought?
4. How might you describe grieving the loss throughout this time?	(a) Ways in which you coped with the loss (b) Difficulties in the process
5. What role did your family environment have in the grieving process?	(a) Ways in which it enabled (b) How it made it difficult
6. What role did your partner have in the grieving process?	(a) Ways in which they enabled (b) Ways in which they made it difficult
7. What role did your social and work environment have in this process?	(a) Ways in which it enabled (b) How it made it difficult
8. What role did your religious/spiritual beliefs and practices play in this process?	(a) Ways in which it enabled (b) Ways in which it made it difficult
9. What aspects do you think affected your grieving process?	(a) Physical illnesses (b) Mental disorders
10. How do you think about your future life project?	

**Table 3:** Verbatim quotes related to “Contextual Aspects of Perinatal Loss”.

Codes	Verbatim Quotes
Gestational Age	“..they couldn’t take my baby out […] it was a whole trauma, I’ve only just started feeling better physically a month ago.” (P6)
	“It was very traumatic in terms of removing the baby from my womb, the curettages were traumatic, with lots of bleeding and pain.” (P5)
Knowledge of Cause of Death	“..generally, in the first trimester, there’s no clear cause, I mean, generally, it’s due to a baby’s alteration.” (P2)
	“An apparent respiratory arrest, also caused by sepsis, you know? There was an infection, and the liver wasn’t functioning well, and the pulmonary hypertension caused oxygen desaturation...” (P4)
History of Previous Losses	“And the second time, I told the Virgin when I went to Chile, if I get pregnant again, I will have it. I don’t want to go through this again.” (P5)
Effects of Perinatal Loss	“There were moments when the room was closed, and there were moments I’d enter and see everything, her clothes, everything, and now the room is open […] it’s been hard. I haven’t been able to sell her bed; I don’t want to sell it because I say it’s hers...” (P4)
	“He told me he was going to throw everything away, but I asked him not to because I knew I would eventually want to remember everything, but it wasn’t the time to remember...” (P3)
	“I waited many years before trying for another pregnancy. I just had a baby four months ago […] I was still fearful, even though I’d undergone tests and everything was fine...” (P1)
	“..and of course, going through a pregnancy after a loss has its challenges, right? There’s more anxiety, fear that the same thing will happen.” (P2)
	“..it wasn’t like the first pregnancy; the first pregnancy was all joy at the beginning. This pregnancy was marked by fear that it might happen again, fear of losing the baby again, constant fear […] and that fear lasted until the end...” (P3)

**Table 4:** Verbatim quotes related to “Emotions associated with perinatal loss”.

Code	Verbatim Quotes
Sadness	“..it was hard, wasn’t it? Losing him.” (P1)
	“..it’s a very difficult moment with many emotions, there is sadness, grief, anger, frustration, yes.” (P2)
	“It will always hurt, right? It could be 1 year, 2 years, the pain will not go away […] and it hurts because you know it will never pass…” (P4)
Anger	“..it makes me very angry because, I mean, people might think […] like, you’ll say, ‘I’ll forget and just have another one,’ or as if you’ll ever forget your child. No.” (P4)
Anxiety	“..I would wake up at 3 in the morning, and from then until the next day, I couldn’t sleep, and I felt anxious and cried…” (P6)
Social Withdrawal	“..I tried not to go out much, socially, because wherever I went, people would ask, ‘How’s your belly?’…” (P1)
	“..the last thing you want is to be with people or have them talk to you […] I just wanted to be alone…” (P4)
Guilt	“..there’s guilt; you feel like it was you, that something failed in you. It can’t be that my body killed my own child—that’s the first thing I thought.” (P5)
	“..the first thing you think is, ‘What did I do wrong?’ I went to work every day; I wasn’t scared of COVID. I mean, in your mind, it’s your fault…” (P7)
Envy or rejection of pregnant women	“..it was because she had been able to have her baby, and I hadn’t […] these feelings of envy come up […] even rejection of people who have their baby […] When I saw a pregnant woman, I felt jealousy—overwhelming jealousy that turned into anger…” (P3)
	“..I had two pregnant friends, and you don’t want to see the other pregnant, right? Because it hurts—it hurts to accept that mine was lost, so it’s hard, it’s hard to see pregnant women…” (P5)
Emotional Numbing	“..it was like I couldn’t feel anything; I was emotionally numb at that moment.” (P3)

**Table 5:** Verbatim quotes related to “Bond with the Baby”.

Codes	Verbatim Quotes
Timing of the Pregnancy	“It was a wanted baby, filled with much joy, hope, and happiness.” (P2)
	“As I said, it was highly anticipated. The moment I took the test, it was something very joyful for both of us…” (P3)
Mother’s Expectations with the Pregnancy	“..they were going to bring joy to the house, and we had gotten excited.” (P1)
	“..as soon as we found out, we told our parents, and everyone was happy [...] we were already starting to plan our lives for after the baby was born, even choosing a name.” (P3)
	“..we had to wait a few more weeks. They confirmed that the baby was there [...] I was very hopeful; I have an older son who is 7 years old, and he was the most excited among all of us [...] my family was also eagerly waiting…” (P6)
Mother’s Life Project	“I am happy, fulfilled with my two children [...] I am enjoying them to the fullest, feeling happy with them, complete, and well, we’ll see what happens after this pandemic to decide if I think about a third.” (P1)
	“The truth is, I can’t imagine a future without children [...] my life plan is to find more balance, not put all the importance on work [...] start a family next year...” (P7)

**Table 6:** Verbatim quotes related to “Support and Silence in face of Loss”.

Code	Verbatim Quotes
Family Support	“..they kept me distracted with their love. I think it’s a way to realize that you also have other kinds of affections around you that also strengthen you…” (P1)
	“..she has been taking care of my little son, supporting me, supporting my husband. My mother played a very important role, as did my husband and his family. My sister-in-law came over […] she helped me during the postoperative period…” (P6)
	“..they think, oh, she’s young, she’ll have kids […] I don’t know why they never ask me; maybe it’s because they feel uncomfortable, they don’t know how to approach or ask…” (P4)
	“..it hurt them so much too […] so they wouldn’t suffer, I put on a strong front…” (P5)
Partner’s Support and Closeness	“He always supported me […] he respected my process, and I respected his […] I always try to be there for him, to listen…” (P4)
	“..thank God I can cry with him, count on him…” (P5)
	“Even now, my husband and I still talk about it […] it strengthened our relationship, didn’t it? Yes, he was a great emotional support; he was always there.” (P3)
Social Environment: Distance, Silence, and Support	“..my coworkers didn’t talk about it either, nor did they ask me. After the first two days of my return, no one mentioned it again. So, they neither helped nor hindered my process.” (P3)
	“It’s like this topic is avoided, but no one talks about Martina either […] I think it’s something about me, not wanting anyone to forget her…” (P4)
	“..friends have been there for us, his friends, my friends […] my clients, even my husband’s bosses. They’ve supported us a lot…” (P6)
	“..I spoke to a friend who had also experienced a loss many years ago. It’s a friend I hadn’t spoken to in years, but she was super empathetic and supportive…” (P7)
Cultural Environment: Insensitivity and Silence About Perinatal Loss	“..none of my partner’s family here in Bolivia knew about the pregnancy, and I decided not to share the loss with them. A very specific reason for this is the lack of awareness about pregnancy loss in Bolivia…” (P2)
	“..I think these grief processes are very internal. I believe they can’t be externalized much because people don’t understand them.” (P3)
	“..people make so many comments; they’re cruel without realizing it. There’s no word, no word that can comfort anyone.” (P4)
	“..so often, the response is: ‘But you’ll have another one’ […] those comments don’t help at all; they make things worse. It’s crucial to acknowledge the loss and just listen […] accompany the grief and recognize it as a process…” (P2)
	“..people tend to keep their distance, they don’t know what to do, or they act as if nothing happened.” (P7)

## Data Availability

Data will be made available upon request to the corresponding author.
